# Denoising diffusion probabilistic models for 3D medical image generation

**DOI:** 10.1038/s41598-023-34341-2

**Published:** 2023-05-05

**Authors:** Firas Khader, Gustav Müller-Franzes, Soroosh Tayebi Arasteh, Tianyu Han, Christoph Haarburger, Maximilian Schulze-Hagen, Philipp Schad, Sandy Engelhardt, Bettina Baeßler, Sebastian Foersch, Johannes Stegmaier, Christiane Kuhl, Sven Nebelung, Jakob Nikolas Kather, Daniel Truhn

**Affiliations:** 1 Department of Diagnostic and Interventional Radiology, University Hospital Aachen, Aachen, Germany; 2 Physics of Molecular Imaging Systems, Experimental Molecular Imaging, RWTH Aachen University, Aachen, Germany; 3 Ocumeda GmbH, Munich, Germany; 4 Artificial Intelligence in Cardiovascular Medicine, University Hospital, Heidelberg, Germany; 5 Department of Diagnostic and Interventional Radiology, University Hospital Würzburg, Würzburg, Germany; 6 Medical Clinic I, University of Mainz, Mainz, Germany; 7 Institute of Imaging and Computer Vision, RWTH Aachen, Aachen, Germany; 8 Department of Medicine III, University Hospital Aachen, Aachen, Germany; 9 Else Kroener Fresenius Center for Digital Health, Medical Faculty Carl Gustav Carus, Technical University Dresden, Dresden, Germany; 10 Division of Pathology and Data Analytics, Leeds Institute of Medical Research at St James’s, University of Leeds, Leeds, UK; 11 Medical Oncology, National Center for Tumor Diseases (NCT), University Hospital Heidelberg, Heidelberg, Germany

**Keywords:** Computer science, Magnetic resonance imaging, Three-dimensional imaging, Computed tomography

## Abstract

Recent advances in computer vision have shown promising results in image generation. Diffusion probabilistic models have generated realistic images from textual input, as demonstrated by DALL-E 2, Imagen, and Stable Diffusion. However, their use in medicine, where imaging data typically comprises three-dimensional volumes, has not been systematically evaluated. Synthetic images may play a crucial role in privacy-preserving artificial intelligence and can also be used to augment small datasets. We show that diffusion probabilistic models can synthesize high-quality medical data for magnetic resonance imaging (MRI) and computed tomography (CT). For quantitative evaluation, two radiologists rated the quality of the synthesized images regarding "realistic image appearance", "anatomical correctness", and "consistency between slices". Furthermore, we demonstrate that synthetic images can be used in self-supervised pre-training and improve the performance of breast segmentation models when data is scarce (Dice scores, 0.91 [without synthetic data], 0.95 [with synthetic data]).

## Introduction

Deep learning (DL) in medical imaging has become ever more relevant. A prototypical problem that DL can solve involves classifying an image, i.e., the condensation of the high-dimensional data within the image down to a single class. The reverse action, i.e., generating images from low-dimensional non-image inputs, is hardly explored, yet, it has enormous potential. Synthetic images can be used to share protected data between sites, for educational purposes, or to predict the progression of diseases in radiographs^1,2^. However, the mentioned studies have primarily been performed on two-dimensional (2D) images^3^. Yet, modern medicine's most important diagnostic imaging modalities, i.e., magnetic resonance imaging (MRI) and computed tomography (CT), yield 3D data. Thus, the concentration on 2D images ignores potentially useful data dimensions that could improve their evaluation. Hence, methods to generate synthetic 3D data are needed.

Previous studies have employed generative adversarial networks^4,5^ (GANs). However, this technique has severe limitations: first, training these models is complex, and mode collapse is a common problem^6^, meaning that the neural network cannot generate diverse samples. Second, the diversity of images generated by these models is limited even if no mode collapse occurs^7^. Third, GANs and similar models focus on the image domain only, and generating images from text or vice versa is not straightforward. In contrast, diffusion models have succeeded in the non-medical domain by generating a wide diversity of images and linking imaging and non-imaging data^8,9^. Despite their superior performance, diffusion models have not been systematically used for 3D image generation in medicine (see below).

In this study, we examine if there is potential for diffusion models in medicine to generate 3D data. We present a new architecture for a diffusion model that works on the latent space of three-dimensional medical images. We train this architecture on four publicly available datasets comprising CT and MR images from variable anatomic regions, i.e., brain MRI, chest CT, breast MRI, and knee MRI. We investigate if the images appear plausible to medical experts and quantify their diversity. Finally, we demonstrate a potential real-life application of these synthetic images in clinical contexts. We investigate whether pre-training on synthetic images improves segmentation models in limited-data settings. We make our code publicly available to foster further research and establish a baseline for future reference.

## Related work

Latent diffusion models have recently gained attention due to their success in generating high-quality images in the non-medical domain by linking image data with text^10^. There is continued interest in translating the concept to the medical domain since such models could be used for various tasks, including data anonymization and augmentation, education and training, and discovering new morphologic associations^11^.

3D image data are generated by CT and MRI (i.e., the preeminent cross-sectional image modalities) and are more challenging to process than 2D image data. While there is some work that applied diffusion models to generate e.g. 3D point clouds, their application to 3D medical data is limited^12^. To our best knowledge, only one concurrent study used diffusion models in the latent space of a variational autoencoder^13^ (VAE) to generate 3D MRI data based on an extensive database of brain scans^14^. However, several groups have worked on utilizing generative adversarial networks to generate 3D data^4,15^. In contrast, our approach can be seen as an extension of latent diffusion models. We append diffusion probabilistic models to the latent space of a Vector Quantized Generative Adversarial Network^16^ (VQ-GAN) to generate high-resolution 3D images. The VQ-GAN model relies on methods that leverage the power of a discriminator and a vector quantized latent space, producing higher-quality image reconstructions, in particular less blurry images, compared to conventional variational autoencoders^16–18^. Producing high-quality reconstructions is crucial as the quality of the images (generated by modeling the latent space using a diffusion model) is largely bottlenecked by the reconstruction performance of the previously learned decoder in the VQ-GAN or VAE model. Additionally, using diffusion models in the latent space has several advantages over using diffusion models directly on the image space of 3D data^19,20^. First, we can reduce the computational resources needed to train the model since it is applied to a compressed latent space with lower dimensionality. Second, while diffusion models excel at generating high-quality images, their sampling speed—especially in comparison to GANs—is relatively low. We can decrease the time needed to generate new samples by sampling images in a lower dimensional latent space. Third, the latent space encapsulates more abstract information about the image, i.e., it covers whole image areas instead of pixel-level information. Thereby, the image is principally accessible to advanced applications such as predicting future image appearances irrespective of image rotation or translation^2^.

The contributions of our study are: (i) We present a method that can generate high-resolution 3D images by learning the structure in the latent space of a VQ-GAN, thereby reducing computational requirements. (ii) The versatility of this method is demonstrated on four publicly available datasets of various modalities and anatomic regions with limited dataset size (n ≤ 1844 images) and we make our code publicly available for everyone to use. (iii) Our model outperforms a well-established reference GAN by generating more diverse images less prone to mode collapse. (iv) Established metrics for quality assessment of synthetic data may fail to assess images like humans^21^. To overcome the limitations of metric-based evaluations, we improve the validity and relevance of these quality assessments by having experienced radiologists assess the quality of the generated images. (v) We demonstrate the potential of the synthetic data generated by our model in low-data settings. By pre-training a Swin-UNETR model in a self-supervised fashion with synthetic images of one institution, we find that this pre-trained model is a good candidate for downstream tasks (such as segmentation) in other institutions. Consequently, the model’s performance is largely improved in low-data regimes compared to models with no pre-training with synthetic data. This finding is particularly relevant in medical imaging, where datasets are typically limited in size.

## Results

### Medical diffusion models can be trained robustly

We trained the diffusion models on publicly available datasets from four different anatomic regions: brain MRI studies from the Alzheimer’s Disease Neuroimaging Initiative (ADNI), chest CT studies from the cancer imaging archive (Lung Image Database Consortium—LIDC), breast MRI studies from Duke University (DUKE), and knee MRI studies from Stanford University (MRNet). These datasets were intentionally chosen to study the capability of our method to generate synthetic data in low-data settings. The four models were thus trained on datasets comprising 998 (brain MRI), 1010 (thoracic CT), 1844 (breast MRI), and 1250 (knee MRI) individual studies.

**Figure 1:**
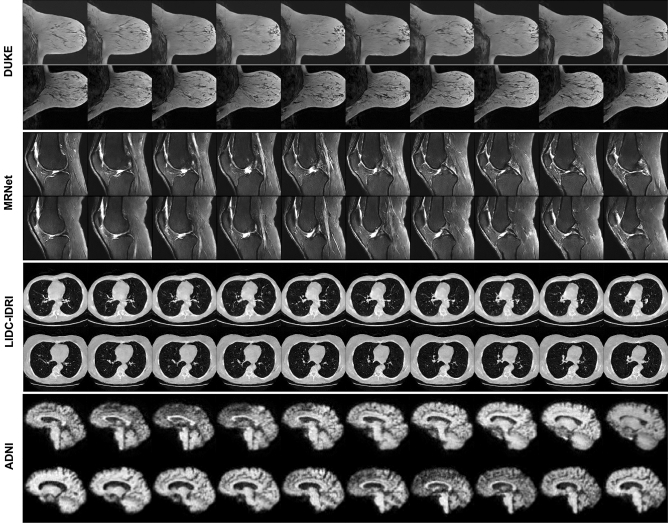
Visualization of synthetic data samples of the four public datasets used to train the medical diffusion model. Each row shows different neighboring z-slices of the same volume. Please note that different image resolutions were used. The breast MRI studies (DUKE dataset, first two rows of images) and the knee MRI studies (MRNet dataset, second two rows) were input to the model at a resolution of 256 × 256 × 32 voxels (height × width × depth). The chest CT studies (LIDC-IDRI dataset, third two rows) had a resolution of 128 × 128 × 128 voxels, while the brain MRI studies (ADNI dataset, fourth two rows) had a resolution of 64 × 64 × 64 voxels.

Even though the datasets were comparatively small, we found that each model converged and generated realistic synthetic images (Fig. 1) without finetuning any hyperparameters to accommodate the different datasets (Supplementary Table S1). In particular, we did not observe mode collapse in any of the training sessions. Additionally, the model architecture could adapt to a wide range of resolutions, covering brain MRI studies with a resolution of 64 × 64 × 64 voxels, chest CT studies with a resolution of 128 × 128 × 128 voxels, and breast MRI and knee MRI studies with an anisotropic resolution of 256 × 256 × 32 voxels. Realistic 3D images could be generated in the four datasets (Fig. 1).

### Medical diffusion models can generate high-quality medical 3D data

**Figure 2:**
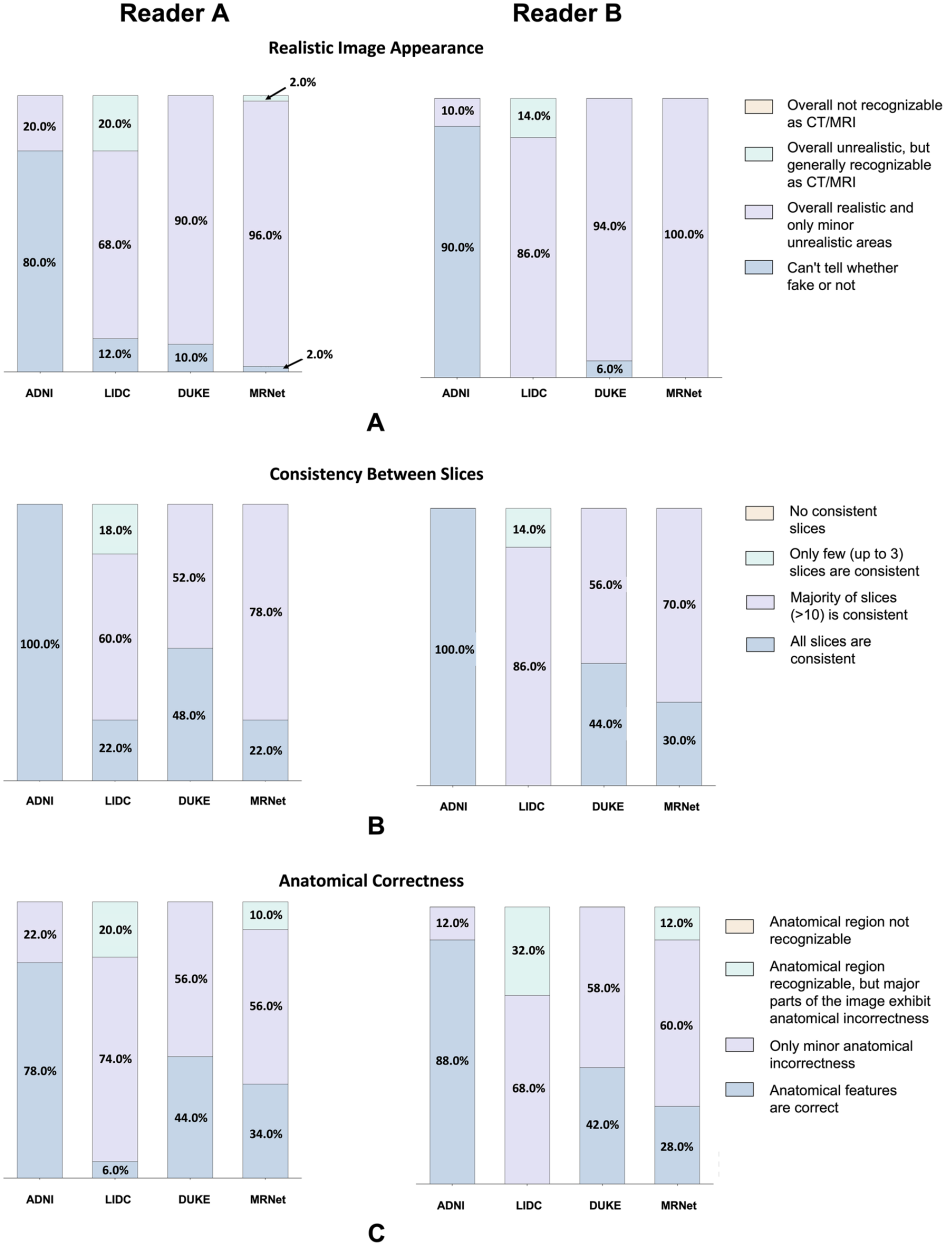
Quantitative evaluation of the image synthesis capabilities on the four datasets used to train the medical diffusion model. Two radiologists with nine and five years of experience in clinical radiology (Reader A and B), respectively, were tasked with evaluating a set of 50 synthetic images from each dataset on a Likert scale. The three categories used to assess the image are "Realistic Image Appearance", “Consistency Between Slices” and “Anatomic Correctness”.

Finding better metrics for assessing the image quality of synthetic images is still an open problem^21^, particularly for medical images where small details may be highly relevant. To overcome this shortcoming of metric-based evaluations, we had human experts rate the synthetic images along three categories: (1) Image quality, (2) slice consistency, and (3) anatomic correctness. Two radiologists with nine years (Reader A) and five years (Reader B) of experience were asked to rate 50 images from each of the four datasets on a Likert scale (Table 1). Reader A rated 189 of 200 images as (at least) largely realistic with only minor unrealistic areas (50/50 for ADNI, 40/50 for LIDC, 50/50 for DUKE, 49/50 for MRNet). 191 of 200 images were considered consistent between most slices (50/50 for ADNI, 41/50 for LIDC, 50/50 for DUKE, and 50/50 for MRNet). 185/200 images were assessed as exhibiting only minor or no anatomic inconsistencies (50/50 for ADNI, 40/50 for LIDC, 50/50 for DUKE, and 45/50 for MRNet). Reader B scored similarly (Fig. 2). Consequently, our architecture can generate synthetic images that appear realistic to radiologists.

**Table 1:** Overview of the categories presented to the radiologists when tasked to assess the quality of the synthetic images.

	Option A	Option B	Option C	Option D
Realistic image appearance	Overall not recognizable as CT/MRI	Overall unrealistic, but generally recognizable as CT/MRI	Overall realistic and only minor unrealistic areas	Can’t tell whether fake or not
Consistency between slices	No consistent slices	Only few (up to 3) slices are consistent	Majority of slices (> 10) are consistent	All slices are consistent
Anatomic correctness	Anatomic region not recognizable	Anatomic region recognizable, but major parts of the images exhibit anatomic incorrectness	Only minor anatomic incorrectness	Anatomic features are correct

The radiologists were asked to select one of four options for each category.

### The dimension of the latent space is important for high-quality image generation

**Figure 3:**
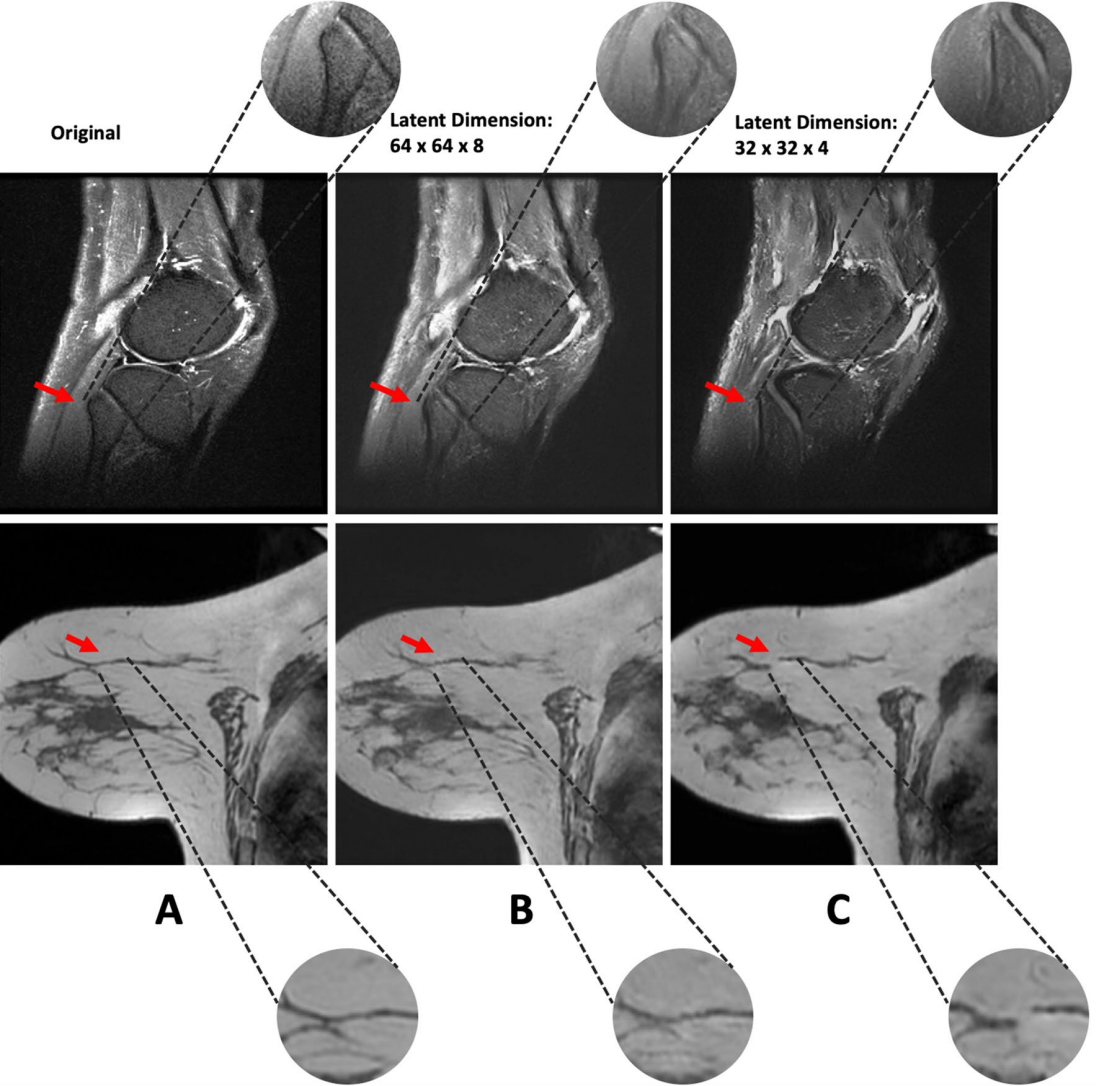
Comparison of the reconstruction quality of the VQ-GAN autoencoder when using different compression factors for two different samples. A latent dimension of 64 × 64 × 8 (i.e., a compression factor of 4 in each dimension, (**B**)) allows for a detailed reconstruction of the original image (**A**) by largely upholding anatomic consistency. A compression factor of 8 (i.e., a latent dimension of 32 × 32 × 4, (**C**)) distorts the fibular bone structure in knee MRI studies (upper row of images) and the fibroglandular tissue structure in breast MRI studies (lower row image). Adjacent, please find zoomed close-up views of the anatomic region of interest.

To analyze the latent dimension's effect on the quality of the synthesized images, we trained the VQ-GAN autoencoder with two different compression factors. The compression factor describes the factor by which the original dimension of the image is reduced in each corresponding dimension of the latent space. We found that when compressing each spatial dimension by a factor of 8 (i.e., images of resolution 256 × 256 × 32 have a latent dimension of 32 × 32 × 4), relevant anatomic features were lost (Fig. 3). When training the VQ-GAN autoencoder with a compression factor of 4 (i.e., images of resolution 256 × 256 × 32 have a latent dimension of 64 × 64 × 8), the anatomic features were reconstructed more accurately. For all four datasets, we found that a maximum compression factor of 4 retained accurate anatomic details as assessed by the radiologists in a test set of 20 sample images per dataset.

### Medical diffusion models outperform GANs in image diversity

To compare our diffusion model to an established GAN model, we adopted the approach by Kwon et al.^4^ and chose a Wasserstein GAN with gradient penalty (WGAN-GP) as a baseline. Because we found divergent behavior in training the WGAN-GP with higher-resolution images, we restricted our comparison to the generated brain MR images of resolution 64 × 64 × 64. We compared both models regarding the multiscale structural similarity metric^22^ (MS-SSIM) by averaging the results over 1000 synthetic sample pairs of the same dataset. Higher MS-SSIM scores suggest that the synthetic images generated by each model are more similar, while lower MS-SSIM scores indicate the opposite. We found that the GAN model cannot generate diverse images, as indicated by its high MS-SSIM score of 0.9996, resulting in synthetic images that are often identical. In contrast, the diffusion model achieved a MS-SSIM score of 0.8557, which is closer to the MS-SSIM score of the original data (0.8095). Supplementary Fig. S1 provides representative visual ouputs of both models.

These findings demonstrate that diffusion models can generate more diverse samples representative of the original data distribution. Therefore, these models might be better suited for follow-up projects, e.g., for classification or educational purposes.

### Synthetic data can be used to train neural networks

**Figure 4:**
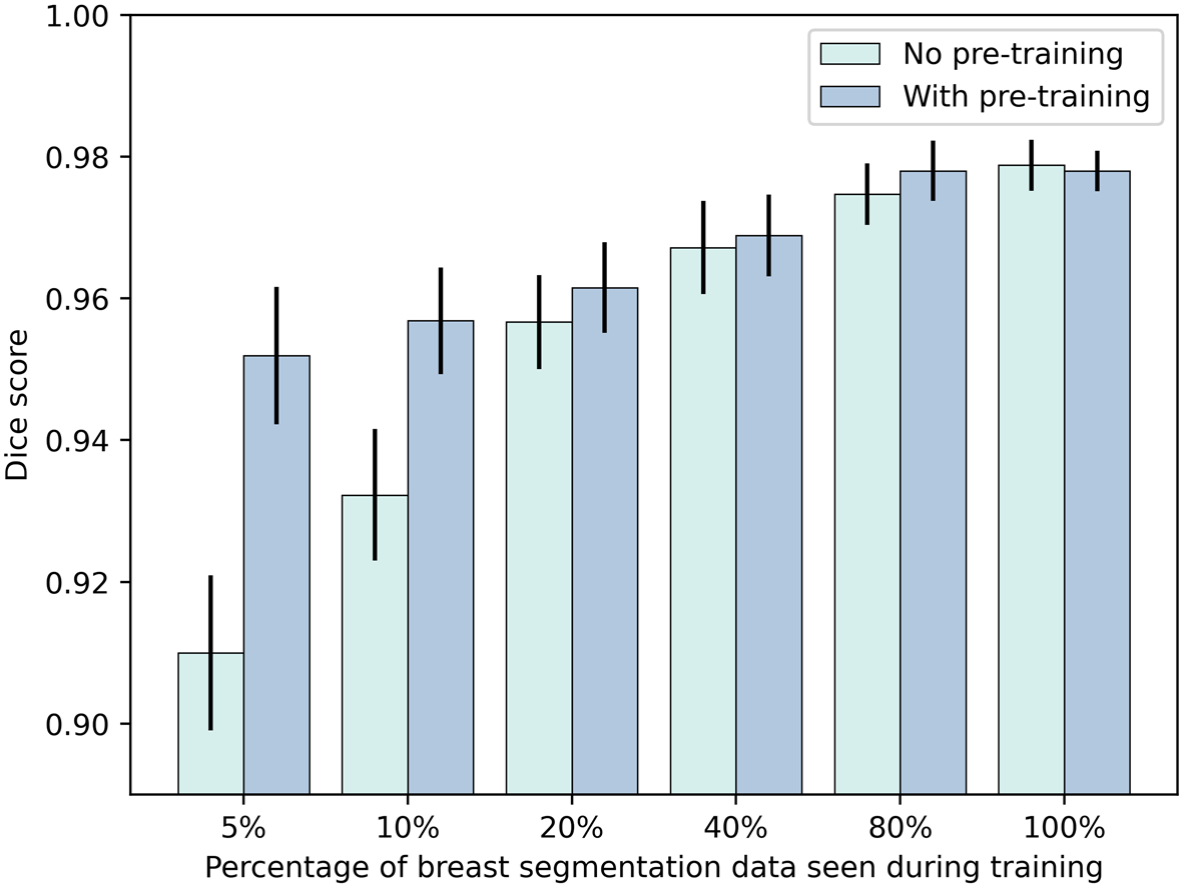
Comparison of the performance of the Swin UNETR in terms of the Dice score for breast segmentation when supplied with synthetic image data (n = 2000) during a self-supervised pre-training setting (“With pre-training”) and when no pre-training (“No pre-training”) is performed. After the optional self-supervised pre-training step, the model is finetuned with 5%, 10%, 20%, 40%, 80%, or 100% of the available internal data with corresponding breast segmentation outlines used as the ground truth (n = 200). We find that the synthetic data generated using a dataset of another institution can substantially improve the Dice score on the internal dataset when only limited data are available for training. Error bars show the standard deviation extracted through bootstrapping (n = 1000).

**Figure 5:**
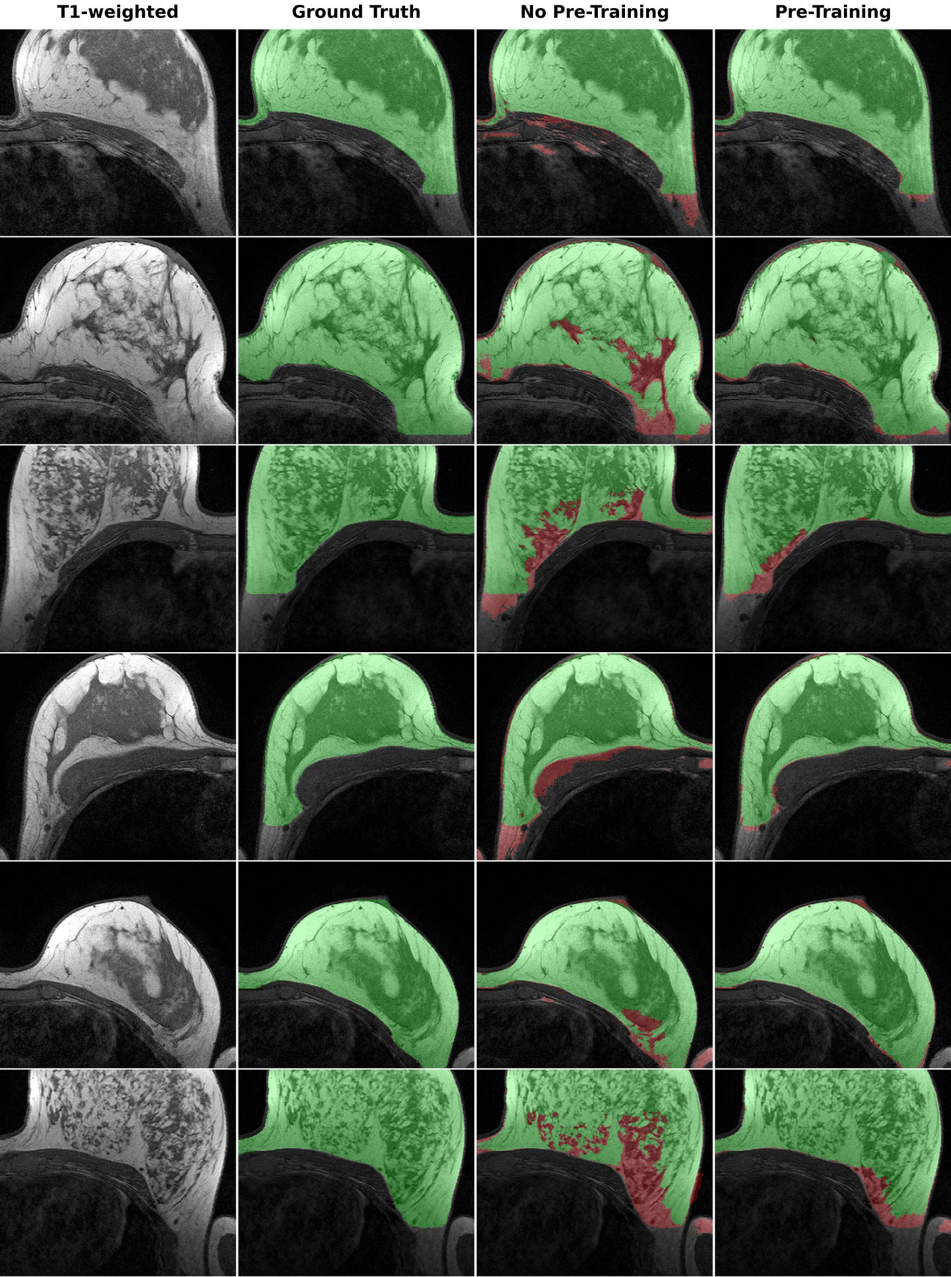
Visualization of the breast segmentation performance for six different studies (rows). The first two columns show the original MR image and the ground truth segmentation of the breast. The third column shows the segmentation of the Swin UNETR neural network when only 5% of the available data from the internal dataset is used during training. The fourth column shows the segmentation of the Swin UNETR when pre-trained in a self-supervised approach using 2000 synthetic images generated using a dataset from another institution and finetuned based on only 5% of the available data from the internal dataset. Green areas denote correctly segmented areas. In contrast, red areas denote deviations from the ground truth.

We evaluated the applicability of synthetic data in a scenario where Institution A seeks to collaborate with Institution B to increase the performance of a neural network without sharing any original data. To this end, we generated 2000 synthetic breast MR images using the diffusion model trained on the DUKE dataset. Since the diffusion model outputs the synthetic images without any ground truth labels, we used the synthetic images to pre-train a Swin UNETR^23^ in a self-supervised fashion. We then finetuned the pre-trained network with the available segmentation data, i.e., the breast MRI studies and the corresponding manual segmentation outlines from Institution B to segment the breast in the MRI studies. To systematically investigate the performance in a limited-data setting, we performed multiple training runs in which we used an increasing portion of the available training data from Institution B, i.e., 5%, 10%, 20%, 40%, 80%, and 100%. For comparison, we trained the same neural network to perform the identical task when no pre-training with synthetic data was performed. Our findings indicate that pre-training with synthetic data from a different institution can substantially improve the segmentation performance, as measured by the Dice similarity coefficient. This improvement became evident in settings where the labeled training data was limited, i.e., Dice similarity coefficients of 0.91 without pre-training and 0.95 with pre-training at 5% available data (Figs. 4 and 5).

## Discussion

As the quality of generative models continues to increase in the non-medical domain, the synthesis of medical data becomes an attainable goal with potential applications in education, data anonymization, data augmentation, and the development of new DL algorithms^2,11^. Diffusion models, in particular, have demonstrated the potential to match human-level performance in image generation^8,9^.

This study presents the first large-scale evaluation of a latent diffusion model applied to MRI and CT data. Such models can generate realistic 3D data that is consistent in its synthetization of continuous 3D structures and capable of accurately reflecting human anatomy. Our findings reveal that our models can achieve robust convergence during training, even when using comparatively small datasets of approximately 1000 sample studies.

In contrast, GANs usually require extensive hyperparameter tuning and large datasets for successful training. GANs often do not converge during training or suffer from mode collapse^6^—a phenomenon we did not observe for our latent diffusion model. More importantly, even if GANs can be trained successfully, our diffusion model can more accurately encompass the diversity of medical images. This finding is particularly relevant for using synthesized images to develop AI models. We showcase a potential medical application of latent diffusion models by utilizing synthesized data to pre-train a segmentation model for human breast MRI studies and show that such pre-training can enhance the robustness of the segmentation models.

Our work has limitations: first, we evaluated our models on comparatively small datasets of about 1000–1800 studies. We intentionally chose such limited study samples to systematically investigate the features of latent diffusion models when limited data is available and distribute computational resources evenly to answer as many scientific questions as possible. Similar models can be expected to generate even more realistic images, potentially at higher resolution, when trained on larger datasets^14^. Second, the generated 3D volumes do not have standard diagnostic image resolution. Limited image resolutions are inherent features of the available public datasets, which may not reflect the state-of-the-art image resolution. Future work on proprietary data may extend our methods to higher image resolutions, which should be possible once more comprehensive computational resources are available. We demonstrate that the compression factor for the latent space is crucial to obtain realistic images. Once large datasets become available for training, e.g., through federated approaches^24^, our findings indicate that image resolution can be scaled up if the compression in the latent space is not too high.

In summary, we have demonstrated that latent diffusion models are superior to GANs in generating synthetic 3D medical data and can form the basis for developing future AI methods on synthetic MRI or CT data.

## Online methods

### Description of dataset

To demonstrate the performance and robustness of the Medical Diffusion model, we trained it on four different publicly available datasets: the MRNet^25^ dataset contains 1250 knee MRI studies from n = 1199 patients, each of which contains a scan along the axial, sagittal, and coronal orientations. To keep our study focussed, we trained our model exclusively on the sagittal T2-weighted sequence with fat saturation. The Alzheimer's Disease Neuroimaging Initiative (ADNI)^26^ dataset contains brain MRI studies from n = 2733 patients. The ADNI was launched in 2003 as a public–private partnership, led by Principal Investigator Michael W. Weiner, MD. The primary goal of ADNI has been to test whether serial MRI, positron emission tomography, other biological markers, and clinical and neuropsychological assessment can be combined to measure the progression of mild cognitive impairment and early Alzheimer's disease. We trained our model on 998 3D magnetization-prepared rapid acquisition with gradient echo (MP RAGE) sequences labeled as cognitively normal. Additionally, we evaluated our model on a breast cancer MRI dataset^27^ (referred to as the DUKE dataset) obtained from 922 breast cancer patients. We used the non-fat saturated T1-weighted sequence from each patient. To demonstrate the generalizability of our Medical Diffusion model, we also trained it to synthesize CT images. To this end, we used 1010 low-dose lung CT studies from 1010 patients of the Lung Image Database Consortium (LIDC) and Image Database Resource Initiative (IDRI)^28^. We also used an internal dataset of 200 non-fat saturated axial T1-weighted sequences of the breast, obtained from 200 patients, with corresponding manual ground truth segmentation outlines of the breast contour. This dataset was included to evaluate the use of synthetic breast MR images in a self-supervised pre-training approach, as detailed below.

### Data pre-processing

Knee MRI studies from the MRNet dataset were pre-processed by scaling the high-resolution image plane to 256 × 256 pixels and applying a histogram-based intensity normalization^29^ to the images. The dataset providers performed this procedure^25^. Additionally, we center-cropped each image to uniform dimensions of 256 × 256 × 32 voxels (height × width × depth). The brain MRI sequences from the ADNI dataset were pre-processed by removing the non-brain areas of the MR images. This procedure was carried out by the dataset providers. To enable comparisons between the diffusion models and GANs, we followed the approach by Kwon et al.^4^ and resized the brain MRI studies to dimensions of 64 × 64 × 64 voxels before feeding the data into the neural network. MR images from the breast cancer dataset were pre-processed by resampling all images to a standard voxel spacing (0.66 mm, 0.66 mm, 3 mm) and then using the corresponding segmentation mask that outlined the breast. The images were split into halves; consequently, the left and the right breast were on separate images. Finally, the images were resized to uniform dimensions of 256 × 256 × 32 voxels. Similarly, the chest CT studies were resampled to a standard voxel spacing of 1 mm in all dimensions. Subsequently, the pixel values were converted to Hounsfield units and the images were center-cropped to dimensions of 320 × 320 × 320 voxels before being resized to 128 × 128 × 128 voxels. Images from all datasets were min–max normalized to the range of − 1 to 1. Additionally, we augmented all datasets by vertically flipping the images during training with a probability of 50%.

### Architecture

Given their substantial size, 3D medical images pose the challenge of finding model architectures capable of generating synthetic images while avoiding computational overburden. We tackle this challenge by using a two-step approach for the Medical Diffusion architecture. First, we encode the images into a low-dimensional latent space and subsequently train a diffusion probabilistic model on the latent representation of the data. Working on the low-dimensional latent space alleviates the problem of limited computational resources. In the following, we provide background information on vector quantized autoencoders, focusing on the VQ-GAN^16^ architecture used to compress the images. Afterward, we introduce the concept of denoising diffusion probabilistic models^30^.

#### VQ-GAN

To encode images into a meaningful latent representation, vector-quantized autoencoders are a viable option as they mitigate blurry outputs commonly encountered in variational autoencoders^17,18^. They map the latent feature vectors located at the bottleneck of the autoencoder to a quantized representation derived from a learned codebook. The VQ-GAN architecture proposed by Esser et al.^16^ is a class of vector quantized autoencoders; here, the image reconstruction quality is further improved by imposing a discriminator loss at its output. More precisely, images are fed into the encoder to construct the latent code $z_{e} \in {\mathbb{R}}^{(H/s) \times (W/s) \times (k)}$, where *H* denotes the height, *W* denotes the width, *k* denotes the number of latent feature maps, and *s* denotes the compression factor. In the vector quantization step, the latent feature vectors are then quantized by replacing each one with its closest corresponding codebook vector *e*_*n*_ contained in the learned codebook *Z*. The image is reconstructed by feeding the quantized feature vectors into the decoder *G*. The learning objective is defined as minimizing the reconstruction loss *L*_*rec*_, the codebook loss *L*_*codebook*_, and the commitment loss *L*_*commit*_. As suggested by Esser et al. in their original publication^16^, we use the perceptual loss as the reconstruction loss and a straight-through estimator to overcome the non-differentiable quantization step. The commitment loss is the mean squared error between the unquantized latent feature vectors and the corresponding codebook vectors. Note that the gradients are only computed for the continuous latent feature vectors to enforce higher proximity to the quantized codebook vectors. The learnable codebook vectors are optimized by maintaining an exponential moving average over all the latent vectors mapped to it. In addition, a patch-based discriminator is used at the output for better reconstruction quality. To extend this architecture to accommodate 3D inputs, we adopted the methodology proposed by Ge et al.^31^ and replaced the 2D convolutions with 3D convolutions. Additionally, we substituted the discriminator in the original VQ-GAN model with a slice-wise discriminator that takes as input a random slice of the image volume and a 3D discriminator that uses the entire reconstructed volume as input. We also follow their approach in adding feature-matching losses to stabilize the GAN training.

#### Diffusion models

Diffusion models are a class of generative models that are defined through a Markov chain over latent variables $x_{1} \cdots x_{T}$^30^. The main idea is that starting from an image *x*_*0*_, the image is continuously perturbed by adding Gaussian noise with increased variance for *T* timesteps. A neural network conditioned on the noised version of the image at timestep *t* and the timestep itself is trained to learn the noise distribution employed to perturb the image. Consequently, the data distribution $p(x_{t - 1} |x_{t} )$ at timestep *t* − 1 can be inferred. When *T* becomes sufficiently large, we can approximate *p*(*x*_*T*_) by the prior distribution ${\mathcal{N}}({\mathbf{0}},{\mathbf{I}})$, sample from this distribution, and traverse the Markov chain reversely. Thereby, we can sample a new image from the learned distribution $p_{\theta } (x_{0} ): = \int {p_{\theta } (x_{0:T} )dx_{1:T} }$. The neural network that models the noise is typically chosen as a U-Net^32^, since the noised input and the denoised output must be the same size. To support 3D inputs, we replaced the 2D convolutions in the original U-Net architecture with 3D convolutions. Each block in the encoder part of the U-Net consists of convolutional layers. These layers downsampled the image using kernels of size 3 × 3 × 1 and only operated on the high-resolution plane of the 3D volume. Subsequently, spatial- and depth-wise attention layers, as proposed by Ho et al^33^, were implemented. The spatial attention layer leveraged the global attention mechanism by computing a key, query, and value vector for every element on the high-resolution image plane, thereby treating the depth dimension as an extension of the batch size. The resultant vectors were combined utilizing the attention mechanism proposed by Vaswani et al.^34^. We introduced a depth-wise attention layer to follow the spatial attention layer. This stage treated the axes of the high-resolution image plane as batch axes, thereby enabling each feature vector on the high-resolution plane to attend to feature vectors on different depth slices. The U-Net's decoder was built similarly, where convolutional layers were applied in each block, followed by spatial- and depthwise attention blocks. In addition, the image was upsampled via transposed convolutions^35^, while skip connections were added to the output of each block.

#### Putting it all together

In the first step, we trained the VQ-GAN model on the whole dataset to learn a meaningful low-dimensional latent representation of the data. As the input to the diffusion model ought to be normalized (i.e., range − 1 to 1), we had to guarantee that the image’s latent representation also fell within this range^30^. Assuming that the vector quantization step in the VQ-GAN model enforced the learned codebook vectors to be close to the latent feature vectors, we approximated the maximum of the unquantized feature representation by the maximum value in the learned codebook. Similarly, we approximated the minimum of the unquantized feature representation by the minimum value in the learned codebook. Thus, by performing a simple min–max normalization on the unquantized feature vectors, we obtained a latent representation with values close to − 1 to 1. These latent representations can subsequently be leveraged to train a 3D diffusion model. By running through the diffusion process in the reverse direction, beginning with noise sampled from a standard Gaussian distribution, we can create latent representations corresponding to new images. The output of this process is quantized using the learned codebook of the previously trained VQ-GAN model and subsequently fed into the decoder to generate the respective image. All models were trained on an NVIDIA Quadro RTX6000 with 24 GB GPU RAM, which took approximately seven days for each model. More details about the training settings for each model can be found in Supplementary Table S1.

#### Self-supervised learning of swin UNETR

To demonstrate the usability of the synthetic data generated by our model, we pre-trained a Swin UNETR^36^ model using self-supervised learning^23^. This method relies exclusively on the generated synthetic data and does not necessitate ground truth labels. We set up the self-supervision task as an inpainting problem, where we masked random patches of the synthetic images followed by random flipping in the horizontal or vertical directions. The neural network was subsequently tasked with reconstructing the missing pixels. We trained the model until convergence utilizing a combination of the L1-Distance and the Multiscale Structural Similarity Index^22^ (MS-SSIM) as loss function, together with the AdamW^37^ optimizer with a learning rate of 1e−3 and weight-decay of 0.01. After training the network, we finetuned the model in a supervised manner, utilizing a different dataset of real images that included manually annotated ground truth labels. A combination of the cross-entropy loss and the dice loss served as the loss function, and the model was trained until convergence using the AdamW optimizer. In this supervised setting, the input images were augmented by applying flipping, affine transformations, ghosting, Gaussian noise, blurring, bias field, and gamma augmentations using the TorchIO framework^38^.

## Supplementary Information

Supplementary Information.

## Data Availability

We conducted the experiments on publicly accessible data to allow for reproducibility assessment and validation by others. Only the breast segmentation model used to test the medical applicability of synthetic data relied on in-house data—the dataset is available upon reasonable request from the corresponding author. The LIDC-IDRI (https://wiki.cancerimagingarchive.net/pages/viewpage.action?pageId=1966254) and the breast MRI (DUKE, https://wiki.cancerimagingarchive.net/pages/viewpage.action?pageId=70226903) datasets are available at the cancer imaging archive (TCIA)^39^. The ADNI dataset is freely available at the Image and Data Archive (IDA, https://adni.loni.usc.edu/^40^. The MRNet dataset is directly available from the dataset providers (https://stanfordmlgroup.github.io/competitions/mrnet/)^25^.

## Code availability

The code is publicly available on GitHub: https://github.com/FirasGit/medicaldiffusion.
