# Supplementary material for: Denoising diffusion probabilistic models for 3D medical image generation
Source: Sci Rep. 2023 May 5;13:7303. doi: 10.1038/s41598-023-34341-2 (PMC10163245; doi:10.1038/s41598-023-34341-2)
Supplement: Supplementary file 1 — Supplementary Information. [file 41598_2023_34341_MOESM1_ESM.docx]

Denoising Diffusion Probabilistic Models for 3D Medical Image Generation

Authors: Firas Khader M.Sc. (1), Gustav Müller-Franzes M.Sc. (1), Soroosh Tayebi Arasteh M.Sc. (1), Tianyu Han M.Sc. (2), Christoph Haarburger PhD (3), Maximilian Schulze-Hagen MD (1), Philipp Schad MD (1), Sandy Engelhardt Prof. (4), Bettina Baeßler Prof. (5), Sebastian Foersch MD (6), Johannes Stegmaier Prof. (7), Christiane Kuhl Prof. (1), Sven Nebelung MD (1), Jakob Nikolas Kather^†^ Prof. (8, 9, 10, 11), Daniel Truhn^†,*^ MD (1)

*corresponding author
^†^equal contribution

# Supplemental Material

**Supplementary Table 1.** Hyperparameters used for training our Medical Diffusion model.

|  | **MRNet** | **ADNI** | **Breast MRI** | **LIDC-IDRI** |
| --- | --- | --- | --- | --- |
| Modality | MRI | MRI | MRI | CT |
| No. images | 1,250 | 998 | 1,844 | 1,010 |
| Image Size  (height, width, depth) | 256x256x32 | 64x64x64 | 256x256x32 | 128x128x128 |
| **VQ-GAN** |  |  |  |  |
| Compression rate  (height, width, depth) | (4,4,4) | (2,2,2) | (4,4,4) | (4,4,4) |
| Codebook size | 16,384 | 16,384 | 16,384 | 16,384 |
| Codebook dimensionality | 8 | 8 | 8 | 8 |
| Learning rate | 3e-4 | 3e-4 | 3e-4 | 3e-4 |
| No. training iterations | 100,000 | 100,000 | 100,000 | 100,000 |
| Batch size | 2 | 2 | 2 | 2 |
| **Diffusion Model** |  |  |  |  |
| No. training iterations | 150,000 | 150,000 | 150,000 | 150,000 |
| Learning rate | 1e-4 | 1e-4 | 1e-4 | 1e-4 |
| Batch size | 40 | 10 | 40 | 50 |
| Timesteps [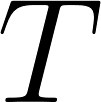](https://www.codecogs.com/eqnedit.php?latex=T#0) | 300 | 300 | 300 | 300 |


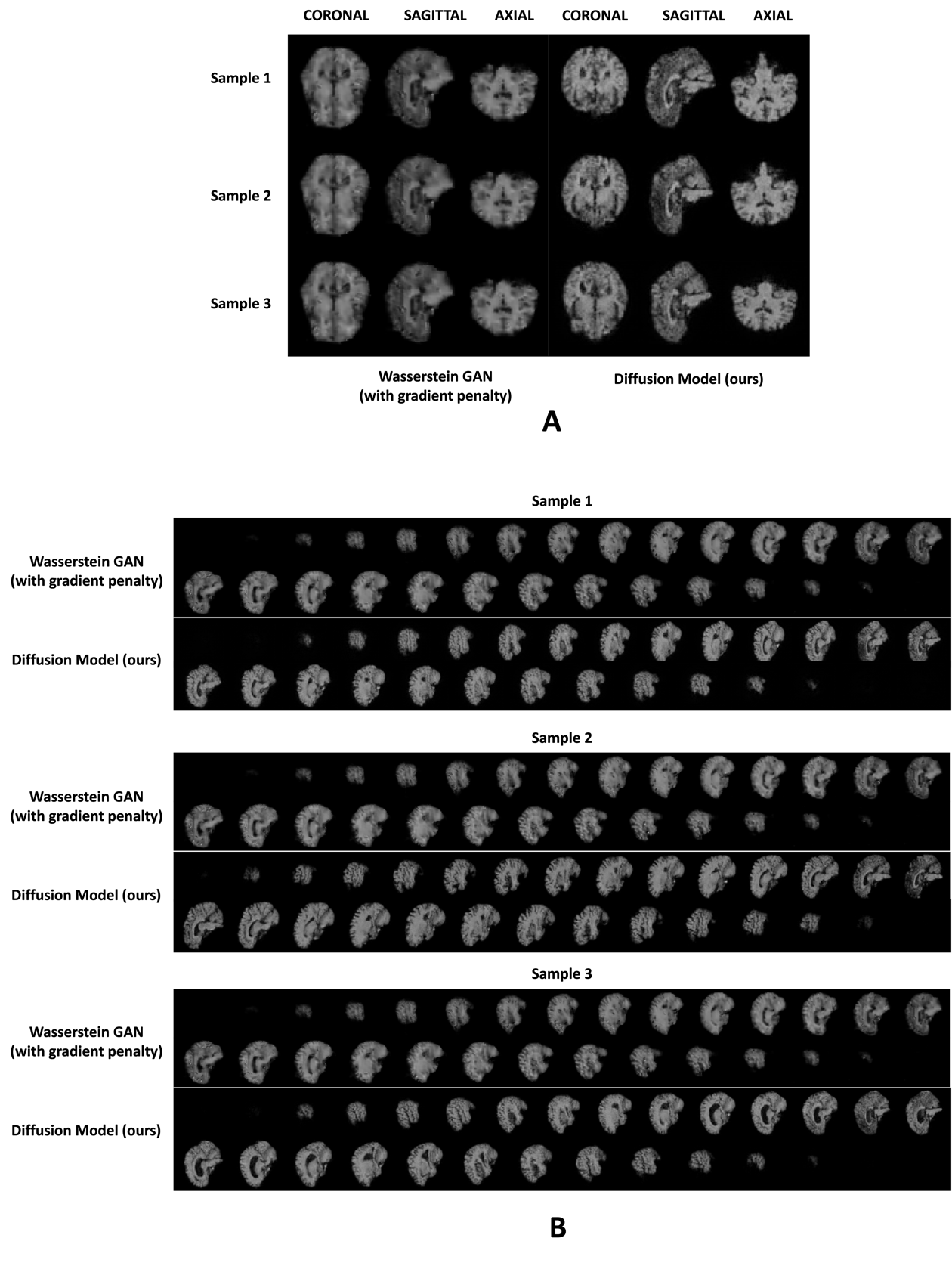


**Supplementary Figure 1:** Comparison of the synthesized images between the GAN-baseline (Wasserstein GAN with gradient penalty) and our approach using diffusion models. (**A)** shows center cut slices for three different samples of the images. We find that the GAN model largely suffers from mode-collapse, resulting in synthetic images with low variance. **(B)** shows individual slices of synthetic images generated by both models. The diffusion model generates more detailed, more diverse and less blurry images compared to the GAN.
